# Quantifying the physical processes leading to atmospheric hot extremes at a global scale

**DOI:** 10.1038/s41561-023-01126-1

**Published:** 2023-02-20

**Authors:** Matthias Röthlisberger, Lukas Papritz

**Affiliations:** Institute for Atmospheric and Climate Science, ETH Zürich, Zürich, Switzerland

**Keywords:** Atmospheric dynamics, Natural hazards

## Abstract

Heat waves are among the deadliest climate hazards. Yet the relative importance of the physical processes causing their near-surface temperature anomalies (𝑇′)—advection of air from climatologically warmer regions, adiabatic warming in subsiding air and diabatic heating—is still a matter of debate. Here we quantify the importance of these processes by evaluating the 𝑇′ budget along air-parcel backward trajectories. We first show that the extreme near-surface 𝑇′ during the June 2021 heat wave in western North America was produced primarily by diabatic heating and, to a smaller extent, by adiabatic warming. Systematically decomposing 𝑇′ during the hottest days of each year (TX1day events) in 1979–2020 globally, we find strong geographical variations with a dominance of advection over mid-latitude oceans, adiabatic warming near mountain ranges and diabatic heating over tropical and subtropical land masses. In many regions, however, TX1day events arise from a combination of these processes. In the global mean, TX1day anomalies form along trajectories over roughly 60 h and 1,000 km, although with large regional variability. This study thus reveals inherently non-local and regionally distinct formation pathways of hot extremes, quantifies the crucial factors determining their magnitude and enables new quantitative ways of climate model evaluation regarding hot extremes.

## Abstract (web summary)

The occurrence of extremely hot days around the globe is the result of a regionally varying mix of physical processes—advective, adiabatic and diabatic warming—that influence upstream air masses, according to an analysis of the backward trajectories of air contributing to hot extremes.

## Main

Atmospheric hot extremes such as the record-shattering heat wave during late June 2021 in the Pacific Northwest^1–3^ (PNW heat wave) regularly claim large numbers of lives, threaten ecosystems and disrupt economic activities^1,4,5^. The number and severity of heat waves will increase globally in response to global warming^6^ with expected severe impacts on human health^7–9^. The socioeconomic relevance of hot extremes is thus more than obvious and mandates the scientific community to provide a quantitative understanding of the relevant physical processes involved in their formation. This process understanding is a prerequisite for evaluating climate models with regard to their ability to realistically reproduce heat-wave characteristics^10^, as well as for developing physically plausible storylines of heat waves and their impacts in a future climate^11–13^.

Existing literature has identified three physical processes that contribute to the formation of atmospheric hot extremes: temperature advection (transport of air from climatologically warmer regions to colder regions^14–16^), adiabatic compression and subsequent warming in subsiding (descending) air^17–20^ and diabatic heating of air near Earth’s surface through surface sensible heat fluxes and turbulent and convective mixing^21–28^. Importantly, however, no consensus has been reached so far about the relative importance of these three processes for atmospheric hot extremes at a global scale^19,25,29^. Rather, individual studies have focused on one particular process^14,15,21,24^, individual case studies^21–24,28^ or hot extremes in selected regions^17–19,26^, leading to diverging results regarding the relative importance of the processes.

In this Article, we quantify the contributions of horizontal temperature advection, adiabatic warming and diabatic heating to near-surface temperature anomalies during the hottest day of each year in 1979–2020 (TX1day events) at each location on the globe. To this end, a new diagnostic based on kinematic air-parcel trajectories is employed. The diagnostic makes use of the Lagrangian temperature-anomaly equation (see Methods for its derivation from the thermodynamic energy equation), which quantifies the change in the temperature anomaly *T*′ along an air-parcel trajectory and allows attributing these changes to the aforementioned processes. In pressure coordinates, the equation reads

1 $$\frac{{{\mathrm{D}}T^\prime }}{{{\mathrm{D}}t}} = - \frac{{\partial \overline T }}{{\partial t}} - v{{{\boldsymbol{\nabla}}}} _{\boldsymbol{h}}\overline T + \left[ {\frac{{\kappa T}}{p} - \frac{{\partial \bar T}}{{\partial p}}} \right]\omega + \left( {\frac{p}{{p_0}}} \right)^\kappa \frac{{{\mathrm{D}}\theta }}{{{\mathrm{D}}t}},$$

where ***v*** is the horizontal wind, ${{{\boldsymbol{\nabla}}}}_{\boldsymbol{h}}$ is the horizontal gradient, $\kappa = {\textstyle{R \over {c_p}}} = 0.286$, *p* is pressure (*p*_0_ = 1,000 hPa), *ω* is the vertical velocity, *θ* is the potential temperature and $\overline T$ is the temperature climatology (see Methods for the formal definition of $\overline T$ used in this study).

Equation (1) allows any temperature anomaly *T*′(***x***,*t*_*X*_) at location ***x*** and time *t*_*X*_ to be decomposed into contributions from the three processes discussed above, provided one knows the backward trajectory (***x***(*t*),*t*) of the air parcel located at ***x*** at time *t*_*X*_, as well as the quantities appearing on the right-hand side of equation (1) along this trajectory. To do so, the terms on the right-hand side in equation (1) are integrated forward in time along the backward trajectory, from the time when the temperature anomaly of this air parcel was last zero (the ‘genesis time’, *t*_g_, of the anomaly *T*′(***x***,*t*_*X*_)) until *t*_*X*_, that is,

2 $$\begin{array}{rcl}T^\prime \left( {{{{\boldsymbol{x}}}},t_X} \right) & = & - \mathop {\int}\limits_{t_g}^{t_X} {\frac{{\partial \bar T}}{{\partial t}}{\mathrm{d}}\tau } - \mathop {\int}\limits_{t_g}^{t_X} {{{{\boldsymbol{v}}}}\nabla _h\overline T {\mathrm{d}}\tau + } \mathop {\int}\limits_{t_g}^{t_X} {\left[ {\frac{{\kappa T}}{p} - \frac{{\partial \bar T}}{{\partial p}}} \right]\omega {\mathrm{d}}\tau }\\ && +\, \mathop {\int}\limits_{t_g}^{t_X} {\left( {\frac{p}{{p_0}}} \right)^\kappa \frac{{{\mathrm{D}}\theta }}{{{\mathrm{D}}t}}{\mathrm{d}}\tau }.\end{array}$$

Terms on the right-hand side of equation (2) denote, respectively, *T*′ arising from changes in the temperature climatology over time, *T*′ due to horizontal advection of the air parcel across climatological temperature gradients, *T*′ generated through vertical motion and *T*′ caused by diabatic processes along the trajectory, including surface sensible heat fluxes and turbulence. These terms are hereafter referred to as seasonality *T*′, advective *T*′, adiabatic *T*′ and diabatic *T*′, respectively. Note that advective *T*′ quantifies *T*′ generated through horizontal advection of air parcels across climatological temperature gradients between *t*_g_ and *t*_*X*_ and is not to be confounded with transport of *T*′ (‘advection of *T*′’). The seasonality *T*′ is usually small on the timescale of the formation of temperature anomalies. The time difference *t*_*X*_ − *t*_g_ is hereafter referred to as ‘Lagrangian age’ of the temperature anomaly *T*′(***x****,t*), and the great circle distance between the location where the anomaly genesis occurred (the ‘genesis point’ ***x***(*t*_g_)) and ***x***(*t*_*X*_) is termed the ‘Lagrangian formation distance’. These two Lagrangian parameters, quantified here, provide essential information about the spatio-temporal formation of *T*′ in hot extremes.

Using European Centre for Medium-Range Weather Forecasts reanalysis ERA5 data^30^, we first apply equation (2) to backward trajectories started on near-surface levels in western North America during the PNW heat wave and then quantify the contributions of advective, adiabatic and diabatic *T*′ to near-surface TX1day anomalies globally.

## The 2021 PNW heat wave

**Fig. 1:**
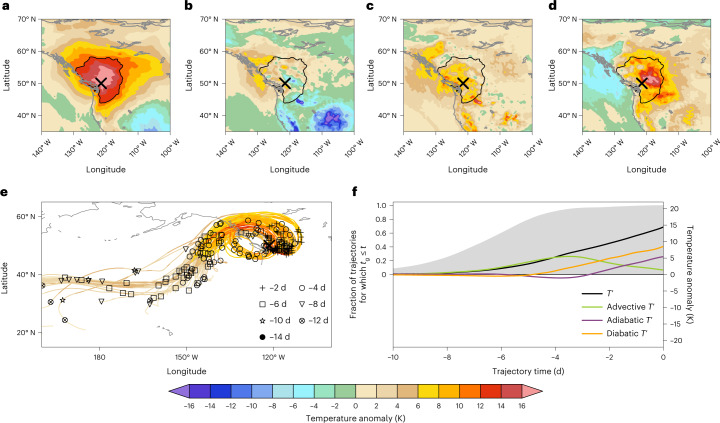
2021 Pacific Northwest heat-wave anomaly decomposition. **a–d**,*T*′ (**a**), advective *T*′ (**b**), adiabatic *T*′ (**c**) and diabatic *T*′ (**d**) averaged for 28–30 June 2021. **e**, The trajectories of air parcels arriving at Lytton (121.5° W, 50.0° N, marked with a black cross in a–e) during the same period. Trajectories are plotted only for trajectory times after the respective anomaly genesis (for trajectory times *t* for which *t*_g_ ≤ *t*) and are coloured according to their respective *T*′ (*t*). **f**, Formation pathway of *T*′, averaged for all trajectories arriving within the heat-wave region during 28–30 June 2021. Grey shading (left *y* axis) denotes the fraction of trajectories for which *t*_g_ ≤ *t* for each trajectory time *t*. Coloured lines show the evolution of *T*′, advective *T*′, adiabatic *T*′ and diabatic *T*′ within these trajectories in Kelvin (right *y* axis). In each trajectory, all terms are set to zero for trajectory times before the respective *t*_g_.

The PNW heat wave peaked during 28–30 June^1^, and near-surface *T*′ averaged across these three days exceeded 12 K in vast parts of western North America (Fig. 1a). The largest three-day mean *T*′ of +18.4 K occurred near Lytton, British Columbia, Canada (cross in Fig. 1a–d), where on 29 June, a daily maximum temperature of 49.6 °C was measured before the town was destroyed in forest fires on 30 June^31^. Evaluating equation (2) for backward trajectories from western North America reveals that all three processes contributed to *T*′, albeit with considerable regional differences in the magnitudes of their contributions (Fig. 1b–d). The advective *T*′ exceeded 6 K west and north of Vancouver Island but was small or even negative where *T*′ was largest. The adiabatic *T*′ contributed positively with larger magnitude than the advective *T*′ (more than 6 K in vast regions in the northwestern United States and British Columbia). Finally, the diabatic *T*′ was clearly dominant in regions of largest *T*′ and exceeded 16 K at some grid points.

**Extended Data Fig. 1:**
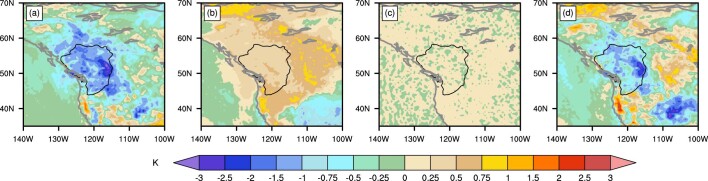
Residual *T*′ during the PNW heat wave and its composition. (**a**) seasonality *T*′, (**b**) *res*1, (**c**) *res*2, and (**d**) *res* = *res*1 + *res*2 + seasonality*T*′. As in Fig. 1a–d all quantities have been averaged from 28 June to 30 June 2021. The black contour in all panels is the same as in Fig. 1a–d. Note the different color scale compared to Fig. 1.

**Extended Data Fig. 2:**
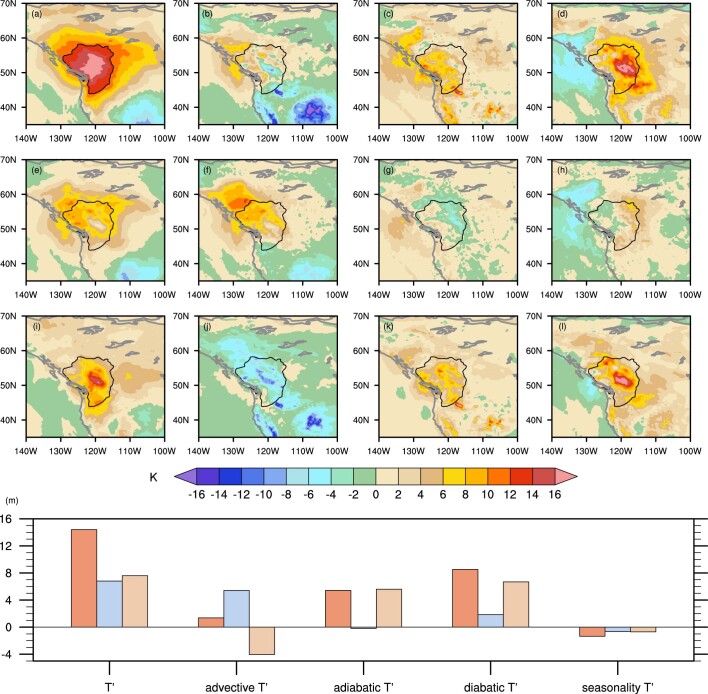
Remote versus local *T*′ generation during the 2021 Pacific Northwest heat wave. Panels (**a–d**) as in Fig. 1. Panels (**e–h**) depict remotely generated *T'*, advective *T'*, adiabatic *T'* and diabatic *T'*, respectively. The quantities have been computed by integrating Equation (1) along the respective trajectories between *t*_*g*_ and *t*_*e*_, whereby *t*_*e*_ is the first trajectory time step during which the respective air parcel was located closer than 500 km to its final position ***x***(t_*X*_). Panels (**i–l**) show locally (that is, less than 500 km distance) generated *T'*, advective *T'*, adiabatic *T'* and diabatic *T'*, respectively, computed as the difference between the top minus middle row. Panel (**m**) shows area weighted averages over the heat wave region (black line in panels a–l) for total *T'* and its contributions (that is, quantities shown in the top row) in red, as well as remotely and locally produced *T'* and its contributions (that is, quantities in the middle and bottom row, respectively) in blue and brown, respectively.

Focusing on backward trajectories started within the heat-wave region (defined here by the 12 K contour in Fig. 1a) at times *t*_*X*_ between 28 and 30 June 2021, we next elucidate the Lagrangian formation pathway of the respective temperature anomalies (see Supplementary Text 1 for more details). The mean age and formation distance of these anomalies were 161 h and 3,834 km, respectively, and most of their genesis points were located over the central North Pacific (Supplementary Fig. 2). After anomaly genesis, the bulk of the air parcels tracked northwards into an amplifying ridge between roughly six and four days before *t*_*X*_, and thereby increased their *T*′ predominantly through advective *T*′ (Fig. 1e,f and Supplementary Video 1). Between four and two days before *t*_*X*_, most of the air parcels moved onshore, which reduced their advective *T*′ (by moving to a climatologically warmer region). At the same time, however, the air parcels also started gaining diabatic *T*′ such that their *T*′ still increased (Fig. 1f). In the final two days, most air parcels gained further diabatic *T*′, descended (generating adiabatic *T*′) and curved anticyclonically towards the south, which consumed nearly all remaining advective *T*′ (Fig. 1f). There is large regional variability in the processes’ relative importance for the final *T*′ (Fig. 1b–d). Nevertheless, averaged across the core heat-wave region, the contributions to *T*′ were, respectively, 59.2% diabatic, 37.7% adiabatic and 9.5% advective, with a residual of −6.3% (mostly seasonality *T*′; Extended Data Fig. 1). In summary, near-surface temperature anomalies during the June 2021 heat wave in the PNW formed through a combination of all three processes, with substantial contributions of remotely generated *T*′, but in particular in regions of maximum *T*′, quasi-locally produced diabatic *T*′ dominated (Extended Data Fig. 2).

## Decomposing TX1day anomalies

**Fig. 2:**
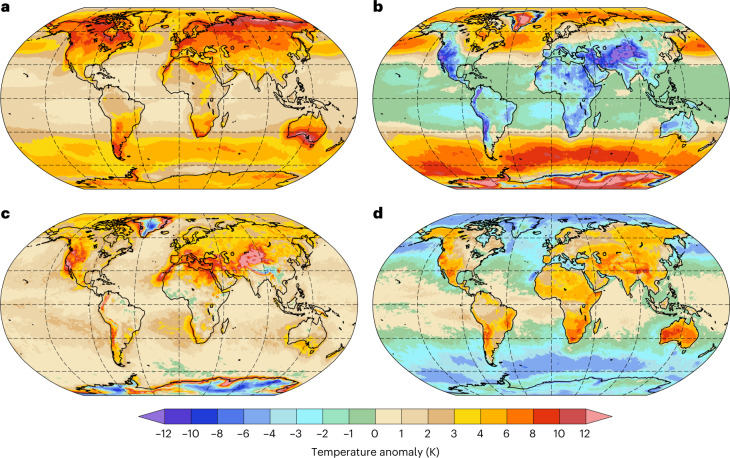
TX1day anomaly decomposition. **a**–**d**, The average temperature anomaly *T*′ during all annually hottest days in 1979–2020 at each grid point (TX1day events) (**a**) and its contributions from advective *T*′ (**b**), adiabatic *T*′ (**c**) and diabatic *T*′ (**d**), averaged over all TX1day events in 1979–2020. Dashed grid lines are shown from 60° S to 60° N every 30° latitude and from 135° W to 135° E every 45° longitude.

We next extend the analysis to a global 42 yr climatology of near-surface temperature anomalies during TX1day events, which reveals geographically strongly varying contributions of the three processes (Fig. 2). Over the mid-latitude storm-track regions, advective *T*′ exceeds the full TX1day anomalies, but is partly offset by negative diabatic *T*′, while the adiabatic *T*′ is small (Fig. 2). Thus, after the respective *t*_g_, air parcels contributing to TX1day events in storm-track regions move polewards across climatological *T* gradients (positive advective *T*′) at near-surface levels (small adiabatic *T*′), and they are cooled diabatically, conceivably through radiation and the cool ocean surface underneath. Similarly, also over the elevated Greenland and Antarctic ice sheets, advective *T*′ dominates, but in contrast to storm-track regions, the adiabatic *T*′ is negative, consistent with orographic ascent (Fig. 2).

**Fig. 3:**
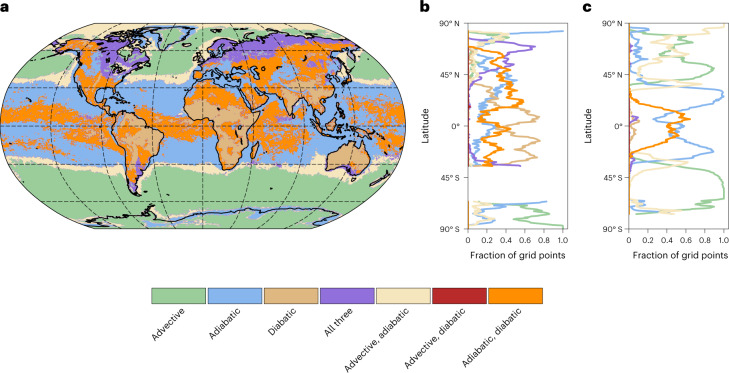
Classifying grid points according to their TX1day anomaly composition. **a**, Green, blue and brown colours indicate regions where one process dominates, whereby a process is deemed dominant if its contribution to the TX1day anomaly is at least twice as large as that of the second most important term. In purple regions, no process dominates, but all three contribute positively to the anomaly. Light brown, red and orange regions also feature no dominant process, but advective *T*′ and adiabatic *T*′ are positive (light brown), advective *T*′ and diabatic *T*′ are positive (red) or adiabatic *T*′ and diabatic *T*′ are positive (orange), with the remaining term being negative. **b**,**c**, The fraction of grid points in each category as a function of latitude for land (**b**) and ocean (**c**) grid points. The lines are masked for latitudes where less than 10% of the grid points are land (**b**) or ocean (**c**) grid points. Dashed grid lines in **a** are drawn as in Fig. 2.

**Extended Data Fig. 3:**
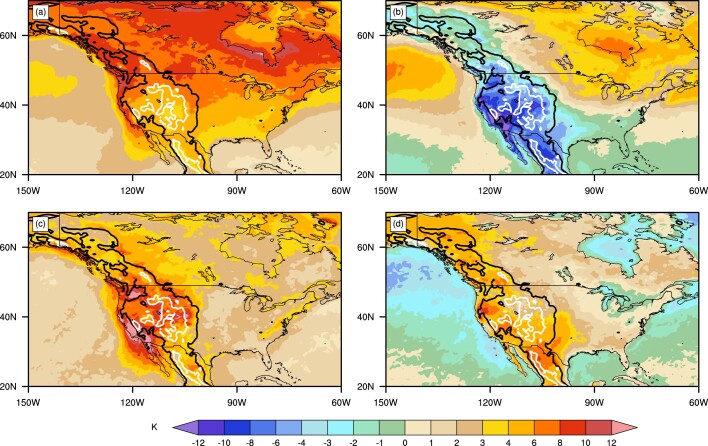
TX1day anomaly decomposition zoom to North America. Same data as in Fig. 2, but zoomed to North America. Shown are (**a**) *T'*, (**b**) advective *T'*, (**c**) adiabatic *T'*, and (**d**) diabatic *T'* for all TX1day events. Black and white contours depict the ERA5 topography at 1000 and 2000 m.a.s.l., respectively.

**Extended Data Fig. 4:**
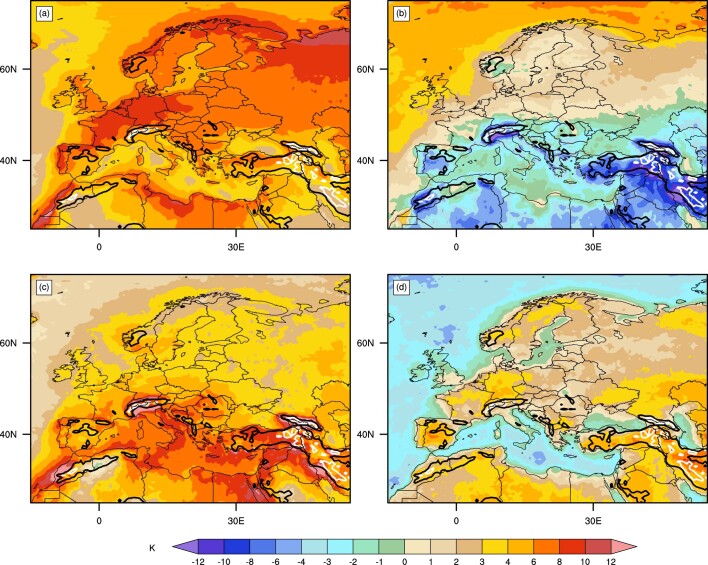
TX1day decomposition zoom to Europe. Same data as in Fig. 2, but zoomed to Europe. Shown are (**a**) *T'*, (**b**) advective *T'*, (**c**) adiabatic *T'*, and (**d**) diabatic *T'* for all TX1day events. Black and white contours depict the ERA5 topography at 1000 and 2000 m.a.s.l., respectively.

**Extended Data Fig. 5:**
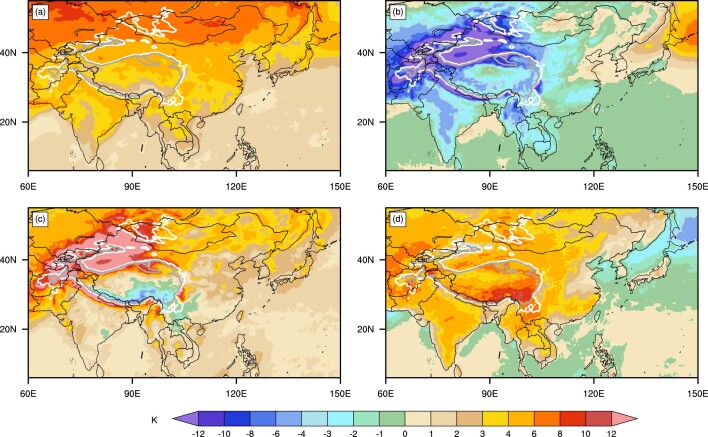
TX1day decomposition zoom to East Asia. Same data as in Fig. 2, but zoomed to East Asia. Shown are (**a**) *T'*, (**b**) advective *T'*, (**c**) adiabatic *T'*, and (**d**) diabatic *T'* for all TX1day events. White, and light grey contours depict the ERA5 topography at 2000, and 3000 m.a.s.l., respectively.

Adiabatic *T*′ dominates in many regions near major mountain ranges, for example, in Nepal, north of the Tibetan Plateau and along the Rocky Mountains (Figs. 2 and 3 and Extended Data Figs. 3–5). Hereby, the largest values of adiabatic *T*′ are not collocated with orographic peaks but rather occur in their vicinity (Extended Data Figs. 3–5). This clear signature of orography in adiabatic *T*′ suggests that downslope winds are a key ingredient of TX1day events in many regions of the world. Furthermore, adiabatic *T*′ dominates in southern Europe and the Mediterranean, where the summer circulation is dominated by anticyclonic conditions and very strong and persistent mid-tropospheric subsidence^32^. Finally, adiabatic *T*′ also dominates TX1day anomalies over subtropical oceans (Fig. 3).

Diabatic *T*′ contributes positively to TX1day anomalies over all land regions except for ice sheets. The largest diabatic *T*′ is in many cases found over dry regions, for example, in central Asia, Mexico, Argentina and western Australia (Fig. 2d), where during TX1day events the availability of soil moisture and thus latent cooling from evaporation is often limited^33^. Over most tropical land regions, the diabatic *T*′ is the dominant contributor (Fig. 3b) and is partially offset by advective *T*′ (Fig. 2). That is, after anomaly genesis, air parcels approach the respective TX1day event location from climatologically colder regions, while they are heated diabatically, conceivably through surface heat fluxes. Globally over the oceans, however, TX1day air parcels are cooled diabatically between the respective anomaly genesis and TX1day event, except in some tropical regions with small positive diabatic *T*′ (Fig. 2d).

**Extended Data Fig. 6:**
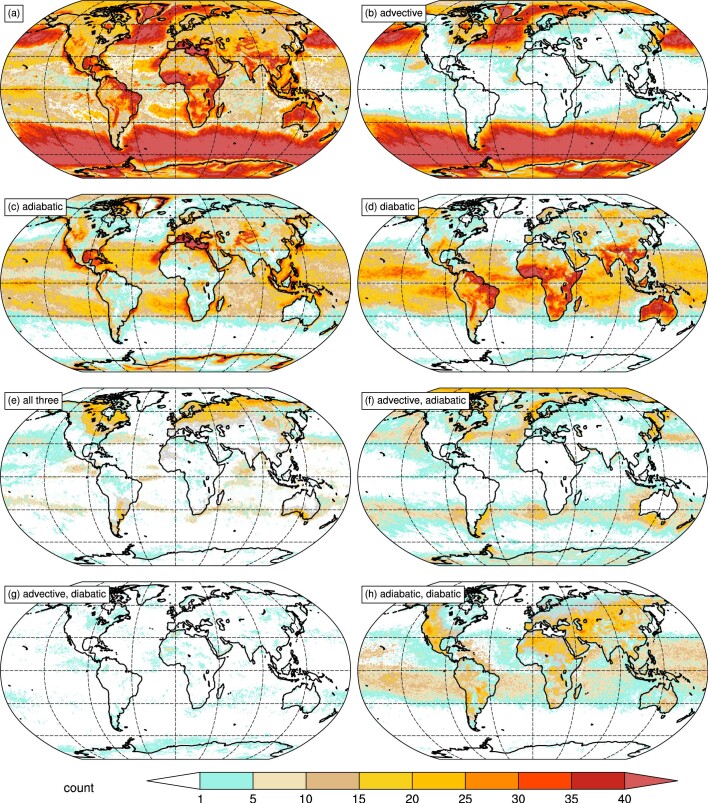
Classifying individual TX1day events. (**a**) The number of individual TX1day events that fall into the category shown in Fig. 3. (**b–h**) Counts of individual TX1day events falling into the category indicated at the top left of each panel. The maximum count that is possible is 42. Dashed grid lines are shown from 60° S to 60° N every 30° latitude and from 135° W to 135° E every 45° longitude.

In many regions, the TX1day anomalies are composed of comparably large contributions from two or even all three processes, without one process clearly dominating. For example, in the Canadian and Eurasian Arctic as well as at the southern tips of South America and Australia, all three processes contribute positively (Figs. 2 and 3). Thus, air parcels contributing to TX1day events in these regions experience anomaly genesis in climatologically warmer regions, then subside and experience net diabatic heating en route to the respective TX1day events. Over several extratropical land regions and over tropical oceans (orange in Fig. 3), the combined effect of adiabatic and diabatic *T*′ generates the TX1day anomalies, while advective *T’* dampens these anomalies (Figs. 2 and 3). Finally, along extratropical coastlines as well as in the high Arctic, TX1day anomalies are composed of positive advective and adiabatic *T*′ and negative diabatic *T*′. Note that there is variability in the contributions of the three processes across TX1day events at any grid point (Extended Data Fig. 6), and hence not all individual events fall into the respective category depicted in Fig. 3.

## Age and formation distance of TX1day anomalies

**Fig. 4:**
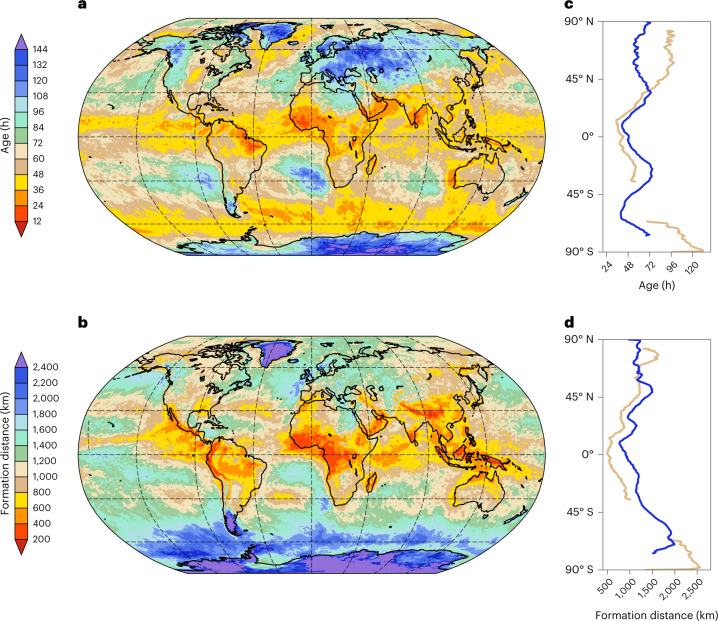
Lagrangian age and formation distance of TX1day anomalies. **a**,**b**, Mean Lagrangian age (**a**) and mean Lagrangian formation distance (**b**) of the TX1day anomalies. **c,d**, Zonal mean of the age (**c**) and formation distance (**d**) for ocean (blue) and land (brown) grid points. The lines in **c**,**d** are masked for latitudes where less than 10% of the grid points are land or ocean grid points. Dashed grid lines in **a,b** are drawn as in Fig. 2.

The age of TX1day *T*′ varies by almost an order of magnitude, from less than a day to roughly a week (Fig. 4a), with a global mean of 60 h. There is no clear land–sea contrast in the age of the anomalies (Fig. 4a), and moreover, there is no apparent universal relationship between the age, the magnitude of TX1day *T*′ and the dominant contributing process for TX1day anomalies. The youngest anomalies are found over tropical West Africa and Brazil (Fig. 4a), where they form due to diabatic *T*′ (Figs. 2 and 3). However, almost equally young TX1day anomalies are found over the Southern Ocean, where advective *T*′ is the dominant contributor. Likewise, the oldest anomalies occur over the Greenland and Antarctic ice sheets (forming through advective *T*′, with negative contributions from adiabatic *T*′) as well as over eastern Europe, where the advective *T*′ is negative and the adiabatic *T*′ is strongly positive.

The formation distance of TX1day anomalies also varies by roughly an order of magnitude, from a few hundred kilometres, for example, in tropical West Africa, to more than 2,000 km over the Greenland and Antarctic ice sheets and the Southern Ocean, with a global mean value of 1,145 km (Fig. 4c,d). Thereby, regions with comparatively old anomalies also feature rather long formation distances and conversely for regions with young anomalies (Fig. 4c,d). A clear exception is the Southern Ocean, where TX1day anomalies form rapidly within less than two days, but on average over distances exceeding 2,000 km, which is in line with generally large wind speeds there.

## Lagrangian information is essential for a quantitative understanding of hot extremes

The lack of consensus about the physical processes that generate atmospheric hot extremes is clearly unsatisfactory given the impacts of these events and the prominent role they play in public and political debates on climate change. This study uses a new quantitative approach to address this research gap and reveals that large near-surface temperature anomalies arise from the accumulation of *T*′ often over large distances rather than just from local weather and/or surface conditions. The Lagrangian approach of this study is in conceptual accordance with previous studies that quantified source regions of heat contributing to past mega heat waves^27,28^ or examined Lagrangian characteristics of heat waves^17–19^. However, it significantly extends these studies by quantifying specifically the contributions of advection, adiabatic compression and diabatic heating to hot extremes.

The global TX1day anomaly decomposition reveals large spatial variability in the relative importance of the three processes and thus demonstrates that none of them—advection, adiabatic compression or diabatic heating—dominates the formation of hot extremes at a global scale. Rather, their relative importance depend on the meteorological intricacies of how large temperature anomalies form in a specific region. These can vary substantially sometimes over just a few hundred kilometres, for example, in the vicinity of complex orography or near coasts. For specific regions, the results of this study qualitatively support the findings of previous studies. For example, in Greenland, long-range transport across climatological temperature gradients was found to be important for melt events^34^ while over tropical land regions, large contributions of anomalously low soil moisture (inducing anomalously strong sensible and turbulent heating) were identified to be particularly important^29^. Moreover, our results underpin the dominant role of advective *T*′ for generating hot extremes in the vicinity of storm tracks^15^.

The mean age and formation distance of temperature anomalies in hot extremes reveal that the formation of these anomalies is inherently non-local and occurs over several days. That is, much like the water vapour that rains out in a heavy precipitation event may have evaporated days before and hundreds or thousands of kilometres away^27,35^, temperature anomalies contributing to hot extremes too are typically built up over several days and over spatial scales of hundreds to thousands of kilometres. These results thus support earlier studies emphasizing the importance of remote processes on mega heat waves^27,28^ and demonstrate that a solely Eulerian perspective on hot extremes is incomplete and inadequate for unravelling the physical causes of such events. We therefore advise considering the Lagrangian characteristics of hot extremes, in particular when evaluating climate models or when pondering about causes of individual events and changing characteristics of hot extremes in a warming climate.

A recent and record-shattering example was the 2021 PNW heat wave^1^. This event’s *T*′ was unprecedented within the 1979–2020 TX1day record, primarily due to exceptional diabatic *T*′ and larger-than-normal advective *T*′ (which is usually negative during TX1day events in the PNW region), while the adiabatic *T*′ was not unusual for hot extremes in this region (Extended Data Fig. 9). The extreme diabatic *T*′ might have been related to dry antecedent conditions^1,36^ and possibly a short-term greenhouse effect arising from comparatively large specific humidity during the event^3^. Furthermore, it suggests a key role for the unusually stable free tropospheric stratification within the atmospheric block during the PNW heat wave^2^, which suppressed deep convection and thereby allowed diabatic *T*′ to accumulate in the boundary layer.

## Implications for hot extremes in a warming climate

Moreover, the results of this study point to two hitherto poorly studied mechanisms for changing magnitudes of hot extremes as the climate warms. First, all else being equal, the projected increase in the tropospheric static stability^37,38^ positively affects adiabatic *T*′ because the third term on the right-hand side in equation (2) increases with increasing climatological stratification (${\textstyle{{\partial \overline T } \over {\partial p}}}$ becomes smaller). Accordingly, a given subsiding air-parcel trajectory in a more stably stratified atmosphere will generate a larger temperature anomaly at the surface. Second, as illustrated for the PNW heat wave, *T*′ contributing to hot extremes over land can form over the ocean (Supplementary Fig. 3). In such cases, the final anomaly is affected by climatological land–ocean temperature contrasts, which, at near-surface levels, are projected to increase as the climate warms^39,40^. Hence, for a given near-surface trajectory, the advective *T*′ will decrease more when moving onshore in a warmer climate, yielding weaker anomalies in such situations. However, these effects will be modulated by circulation changes affecting the trajectories of hot extreme air parcels (more or less vertical, meridional or zonal movement). Therefore, their importance will have to be examined in detail by applying the Lagrangian diagnostic to climate model simulations.

## Methods

### Data

The ERA5^30^ is the latest reanalysis dataset of the European Centre for Medium-Range Weather Forecasts. The data assimilation scheme, model set-up and performance are thoroughly described in ref. ^30^. Here we use ERA5 data at 3-hourly temporal resolution, a horizontal grid spacing of 0.5° latitude by 0.5° longitude and on model levels. The TX1day events in 1979–2020 are identified on the basis of daily mean 2 m temperature at each grid point. The model-level temperature climatology, $\overline T$, is transient, taking the daily and seasonal cycles, as well as the long-term warming trend, into account. Specifically, for each date in the study period, the climatology is computed by averaging over all time steps with the same time of the day in a 21 d window centred around the given calendar time step and within ±4 yr (every $\overline T$ value is thus the average over 21 × 9 = 189 instantaneous values). Moreover,${\textstyle{{\partial \overline T } \over {\partial t}}}$ and ${\textstyle{{\partial \overline T } \over {\partial p}}}$ are computed on the basis of first-order (centred) finite differences.

### Trajectory calculations

At each ERA5 grid point, 15 d (360 h) backward trajectories are started at 00:00, 03:00, 06:00, 09:00, 12:00, 15:00, 18:00 and 21:00 utc during each TX1day event from 10, 30 and 50 hPa above ground level, using LAGRANTO 2.0^41^ (and analogously from the domain shown in Fig. 1a on each day of the June 2021 heat wave). This yields 24 trajectories for each TX1day event and grid point (in total, ~250,000,000 15 d trajectories were calculated for the global TX1day anomaly decomposition). For all figures, the terms in equation (2) have been averaged across trajectories starting from 10, 30 and 50 hPa above ground, which we refer to as ‘near surface’. Each trajectory is stored with a 3-hourly temporal resolution. Along each trajectory (***x***(*t*),*t*), with ***x***(*t*) = (longitude(*t*), latitude(*t*), *p*(*t*)), the following variables are traced: *T*, $\overline T$, *θ*, ${\textstyle{{\partial \overline T } \over {\partial t}}}$ and ${\textstyle{{\partial \overline T } \over {\partial p}}}$.

### Lagrangian temperature-anomaly equation

The thermodynamic energy equation in pressure coordinates reads^42^

3 $$\frac{{{\mathrm{D}}T}}{{{\mathrm{D}}t}} = \frac{{\kappa T\omega }}{p} + \left( {\frac{p}{{p_0}}} \right)^\kappa \frac{{{\mathrm{D}}\theta }}{{{\mathrm{D}}t}}.$$

Defining *T*′ = *T* *−* $\overline T$, inserting this definition in equation (3) and noting that ${\textstyle{{{\mathrm{D}}\overline T } \over {{\mathrm{D}}t}}} = {\textstyle{{\partial \overline T } \over {\partial t}}} + {{{\boldsymbol{u}}}}\nabla \overline T$ yields

4 $$\frac{{{\mathrm{D}}T^\prime }}{{{\mathrm{D}}t}} = - \frac{{\partial \overline T }}{{\partial t}} - {{{\boldsymbol{u}}}}\nabla \overline T + \frac{{\kappa T\omega }}{p} + \left( {\frac{p}{{p_0}}} \right)^\kappa \frac{{{\mathrm{D}}\theta }}{{{\mathrm{D}}t}},$$

where ${{{\boldsymbol{u}}}} = \left( {u,v,\omega } \right)$ and ${{{\nabla}}}$ is the three-dimensional gradient operator. Vertical motion thus creates temperature anomalies through ${\textstyle{{\kappa T\omega } \over p}}$, but at the same time this effect may be cancelled by the vertical advection of the climatological temperature $- \omega {\textstyle{{\partial \overline T } \over {\partial p}}}$ (e.g., when moving from a climatologically colder region aloft to a warmer region below). The net effect of vertical motion on *T*′ is thus given by $\left[ {{\textstyle{{\kappa T} \over p}} - {\textstyle{{\partial \overline T } \over {\partial p}}}} \right]\omega$; that is, subsidence leads to positive *T*′ when the climatological stratification is stable and thus the adiabatic warming ${\textstyle{{\kappa T\omega } \over p}}$ exceeds the vertical advection of the climatological temperature $\omega {\textstyle{{\partial \overline T } \over {\partial p}}}$. Expressing the effect of vertical motion on *T*′ in this way and integrating in time leads to the formulation in equation (2).

### Quantifying the composition of a temperature anomaly

The integrands in the first, third and fourth terms on the right-hand side of equation (2) are evaluated between subsequent trajectory time steps *t* and *t* + Δ*t*. That is, *ω* is computed from the finite difference between the trajectory pressure *p* at *t* and *t* + Δ*t*, and ${\textstyle{{\partial \overline T } \over {\partial t}}}$, *p*, *T* and ${\textstyle{{\partial \overline T } \over {\partial p}}}$ are computed as averages of the respective quantities evaluated at (***x***(*t*),*t*) and (***x***(*t* + Δ*t*),*t* + Δ*t*). For $\left( {{\textstyle{p \over {p_0}}}} \right)^\kappa$ in the fourth right-hand-side term in equation (2), we use *p*_0_ = 1,000 hPa, and ${\textstyle{{{\mathrm{D}}\theta } \over {{\mathrm{D}}t}}}$ is computed as the finite difference between *θ* at (***x***(*t*),*t*) and (***x***(*t* + Δ*t*),*t* + Δ*t*). The integrand in the second right-hand-side term in equation (2) is computed as

5 $$- {{{\boldsymbol{v}}}}\nabla _h\overline T = - \frac{{{\mathrm{D}}\overline T }}{{{\mathrm{D}}t}} + \frac{{\partial \overline T }}{{\partial t}} + \omega \frac{{\partial \overline T }}{{\partial p}},$$

whereby ${\textstyle{{{\mathrm{D}}\overline T } \over {{\mathrm{D}}t}}}$ is also computed from subsequent trajectory time steps analogously to ${\textstyle{{{\mathrm{D}}\theta } \over {{\mathrm{D}}t}}}$, and ${\textstyle{{\partial \overline T } \over {\partial t}}}$ and ${\textstyle{{\partial \overline T } \over {\partial p}}}$ are averages of the respective quantities evaluated at (***x***(*t*),*t*) and (***x***(*t* + Δ*t*),*t* + Δ*t*). This indirect computation of ${{{\boldsymbol{v}}}}\nabla _h\overline T$ is superior to a direct computation based on ***v*** and $\nabla _h\overline T$ at (***x***(*t*),*t*) and (***x***(*t* + Δ*t*),*t* + Δ*t*), because the model levels in the lower troposphere slope strongly near complex topography and therefore make it difficult to accurately compute $\nabla _h\overline T$ in such regions.

**Extended Data Fig. 7:**
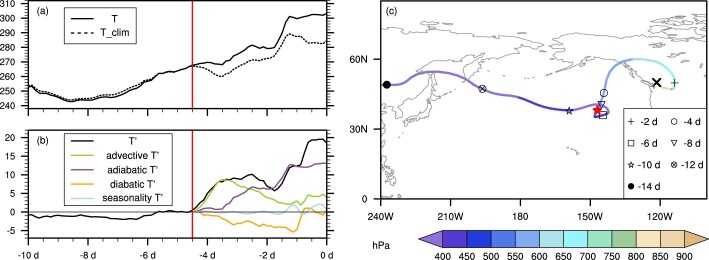
Visualizing the Lagrangian *T*′ decomposition for a single trajectory. (**a**) The *T* and $\overline T$ evolution of the 15-day backward trajectory started at *t*_*X*_ = 18 UTC, 29 June 2021 at 121.5°W, 50°N (Lytton, Canada), in K. The genesis time *t*_*g*_ is indicated in (**a,b**) as a vertical red line, and is identified by following the trajectory backward in time from *t*_*X*_ until the last time step when *T*′ has the same sign as *T*′(*t*_*X*_). (**b**) *T*′ evolution (in K) of the same trajectory. After *t*_*g*_, colored lines in (**b**) show the seasonality (blue), advective (green), adiabatic (purple) and diabatic (orange) contribution to *T*′ at the respective trajectory time step, obtained from integrating Equation (2) from *t*_*g*_ until the respective time step. (**c**) The same trajectory as in (**a,b**), colored by its pressure. The red star marks the genesis location of the final anomaly and the bold black cross locates Lytton, Canada.

**Extended Data Fig. 8:**
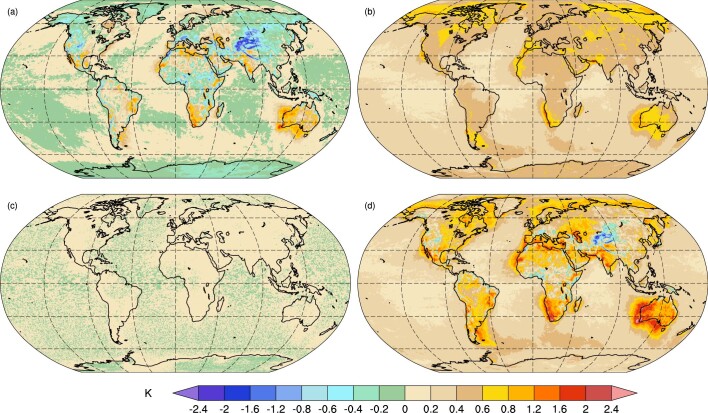
TX1day anomaly decomposition residuals. Shown are (**a**) seasonality *T*′, (**b**) *res*1, (**c**) *res*2, and (**d**) *res* = seasonality*T*′ + *res*1 + *res*2. Note the different color scale compared to Fig. 2. Dashed grid lines are shown from 60° S to 60° N every 30° latitude and from 135° W to 135° E every 45° longitude.

**Extended Data Fig. 9:**
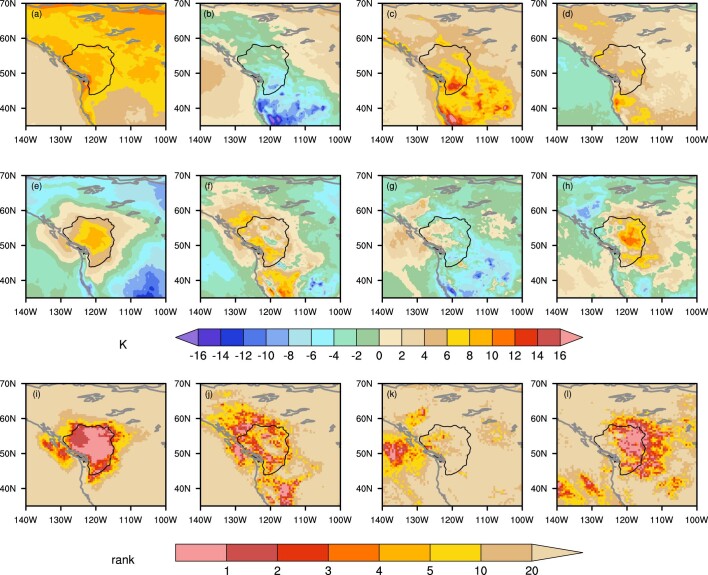
Comparing the June 2021 Pacific Northwest heat wave to TX1day events. (**a–d**) TX1day decomposition as in Fig. 2, but zoomed to the PNW region. Panels (**e-h**) show the difference between *T'*, advective *T'*, adiabatic *T'* and diabatic *T'* during 28–30 June 2021 (same data as depicted in Fig. 1a–d) minus panels (**a–d**) in this figure, that is, positive values in (**e–h**) indicate where the respective quantity was larger during the PNW heat wave than in the local TX1day climatology, and conversely for negative values. Panels (**i–l**) show at each grid point the rank of the respective values in Fig. 1a–d (that is, three day averaged values from the PNW heat wave) within the respective 42 TX1day events in 1979–2020. A rank of 1 indicates that the respective value from the PNW heat wave exceeded all values from TX1day events at the respective grid point.

The genesis time, *t*_g_, of any anomaly *T*′(***x***,*t*_*X*_) is found by following the trajectory from (***x***,*t*_*X*_) backwards in time until the last trajectory time step *t*_g_ where *T*′(***x***(*t*_*g*_),*t*_*g*_) has the same sign as *T*′(***x***,*t*_*X*_) (see Extended Data Fig. 7 for an illustration). Note that *T*′(***x***(*t*_g_),*t*_g_) is never exactly zero and thus constitutes a first numerical residual (res1) arising from applying equation (2) to actual trajectories (Extended Data Fig. 8b). Moreover, in very rare cases where anomalies are older than 15 d (*T*′(***x***(*t*),*t*) is single-signed for the whole 15 d), the age of the anomaly is set to 15 d and ${\mathrm{res}}1 = T^\prime \left( {{{{\boldsymbol{x}}}}\left( {t_X - 15\,{{{\mathrm{days}}}}} \right),t_X - 15\,{{{\mathrm{days}}}}} \right)$. Note that fewer than 0.8% of all TX1day anomalies are older than 15 d (not shown) and res1 is generally small (Extended Data Figs. 1b and 8b).

To decompose any temperature anomaly *T*′(***x***,*t*_*X*_), the terms on the right-hand side in equation (2) are integrated forward in time for each trajectory (***x***(*t*),*t*) from the respective *t*_g_ to *t*_*X*_; that is,

6 $${{{\mathrm{seasonality}}}}\,T^\prime = - \mathop {\int}\limits_{t_{\mathrm{g}}}^{t_X} {\frac{{\partial \overline T }}{{\partial t}}{\mathrm{d}}\tau } ,$$

7 $${{{\mathrm{advective}}}}\,T^\prime = - \mathop {\int}\limits_{t_{\mathrm{g}}}^{t_X} {{{{\boldsymbol{v}}}}\nabla _h\overline T {\mathrm{d}}\tau } ,$$

8 $${{{\mathrm{adiabatic}}}}\,T^\prime = \mathop {\int}\limits_{t_{\mathrm{g}}}^{t_X} {\left[ {\frac{{\kappa T}}{p} - \frac{{\partial \overline T }}{{\partial p}}} \right]\omega {\mathrm{d}}\tau } ,$$

9 $${{{\mathrm{diabatic}}}}\,T^\prime = \mathop {\int}\limits_{t_{\mathrm{g}}}^{t_X} {\left( {\frac{p}{{p_0}}} \right)^\kappa \frac{{{\mathrm{D}}\theta }}{{{\mathrm{D}}t}}{\mathrm{d}}\tau }.$$

Note that the seasonality *T*′ also contains a contribution from the diurnal cycle of $\overline T$. In Extended Data Fig. 7, we visualize the identification of *t*_g_ as well as the Lagrangian *T*′ decomposition for an individual trajectory. The results displayed in Figs. 1a–d and 2 show these terms averaged across all 24 trajectories of all days that are considered in the respective figures.

Finally, exact closure of the temperature-anomaly budget in equation (2) is hindered by a second numerical residual (res2), which arises from numerical inaccuracies in the computations of derivatives in equation (2). This residual can be quantified as res2 = *T*′ (***x***,*t*_*X*_) − res1 − seasonality, *T*′ diabatic*T*′ − advective *T*′ − adiabatic*T*′ − and is found to be negligible (Extended Data Figs. 2c and 8c), even for individual trajectories (not shown). To quantify how well the temperature-anomaly budget in equation (2) is closed by considering only advective, adiabatic and diabatic *T*′, an overall residual res = res1 + res2 + seasonality *T*′ is defined and found to be small for the events of interest in this study (Extended Data Figs. 1d and 8d).

## Online content

Any methods, additional references, Nature Portfolio reporting summaries, source data, extended data, supplementary information, acknowledgements, peer review information; details of author contributions and competing interests; and statements of data and code availability are available at 10.1038/s41561-023-01126-1.

## Supplementary information

Supplementary Information Supplementary Figs. 1–4 and Text 1.

Supplementary Video 1 The Lagrangian formation pathway of the June 2021 PNW heat wave. All trajectories contributing to *T*′ in the heat-wave region (thick black line in all panels) during 28–30 June 2021 have been gridded to a 0.5° latitude by 0.5° longitude grid for all trajectory times after the respective *t*_g_. **a**, *T*′. **b**, Advective *T*′. **c**, Adiabatic *T*′. **d**, diabatic *T*′. The bottom colour bar is valid for **a**–**d**. **e**, The number of trajectories per 0.5° latitude by 0.5° longitude grid cell. **f**, The average pressure of all trajectories in the respective grid box. Values in **a**–**d** have been averaged across all trajectories per grid box. Sea-level pressure contours (thin black contours) and 500 hPa geopotential height contours of 5,700 m and 5,800 m (blue) are shown at valid times indicated on the top right of **e**. These valid times correspond to 12:00 utc, 29 June 2021 (the central time step of the 3 d period analysed here) plus the respective trajectory time.

## Data Availability

The ERA5 data can be downloaded from the Copernicus Climate Service (https://climate.copernicus.eu/climate-reanalysis) and are thoroughly described in ref. ^30^. Data displayed in Figs. 1–4 as well as derived data supporting this study are available from the ETH Research Collection via 10.3929/ethz-b-000571107 ref. ^43^.

## Code availability

The LAGRANTO 2.0 code is publicly available from ref. ^41^. Code to reproduce the main results of this study are available from the ETH Research Collection via 10.3929/ethz-b-000571107 ref. ^43^.
