# Supplementary material for: Quantifying the physical processes leading to atmospheric hot extremes at a global scale
Source: Nat Geosci. 2023 Feb 20;16(3):210–6. doi: 10.1038/s41561-023-01126-1 (PMC10005943; doi:10.1038/s41561-023-01126-1)
Supplement: Supplementary file 1 — Supplementary Figs. 1–4 and Text 1. [file 41561_2023_1126_MOESM1_ESM.pdf]

nature geoscience

Article https://doi.org/10.1038/s41561-023-01126-1

Quantifying the physical processes leading to atmospheric hot extremes at a global scale

In the format provided by the authors and unedited

Supporting Text1: Synoptic evolution of the PNW heat wave and relation to T' formation.

The synoptic evolution of the PNW heat wave has been previously described by Neal et al.[1], we therefore focus here only on synoptic aspects that are particularly relevant for the final composition of the PNW heat wave T' (Supporting Fig. S1) and subsequently describe how T' built up within the PNW heat wave air parcels, based on Supporting Figs S2 and 3 as well as Supporting Anim. 1.

Synoptic evolution of the PNW heat wave

Between 19 and 22 June 2021 a broad and slowly propagating upper-level ridge (labelled "R1" in Supporting Fig. S1a) formed over the eastern North Pacific, with a ridge axis off the North American west coast, while an upper-level cutoff low (L1) was located just off the coast of California. A rapidly deepening cyclone (C2) formed at the southern and eastern fringe of a large pre-existing cyclone (C1) in the gulf of Alaska between 23 and 24 June. The warm-conveyor belt associated with cyclone C2 has been found to be of pivotal importance for a second pulse of ridge-building over the eastern north Pacific, which led to the highly amplified ridge within which the PNW heat wave ultimately occurred[1] (this ridge is labelled "R2" in Supporting Figs. S1 and S2). The ridge R2 amplified between 23 and 27 June, and the ridge axis then slowly crossed the PNW heat wave region between 28 and 30 June. Due to the quasi-stationarity of the highly amplified ridge R2 and the cutoff (L2) to its south, this flow situation constitutes a typical rex-type blocking situation[2].

PNW heat wave T' formation pathway

We next discuss in detail the formation pathway of T' for air parcels that ultimately end up on near surface levels (10, 30 or 50 hPa above ground) within the heat wave region during the 28–30 June 2021 period. For simplicity, we write just "air parcels" hereafter, whenever we refer to this set of air parcels ultimately contributing to the PNW heat wave. For the bulk of the air parcels anomaly genesis occurred along the western flank of ridge R1 (Supporting Fig. S2), between 21 and 24 June (although for some air parcels anomaly genesis occurred over the eastern subtropical Pacific, already a few days earlier, Supporting Fig. S2). However, prior to 22 June, very few of these air parcels featured T' exceeding 6 K and only during the deepening phase of cyclone C2 larger temperature anomalies started appearing within these air parcels (Supporting Anim. 1, e.g., valid time 12 UTC 23 June 2021). These large T' formed within the amplifying ridge R2 as air parcels were advected poleward across climatological temperature gradients (resulting in large positive advective T'). Some of the advective T' was offset by

negative adiabatic T' as the air parcels ascended along their poleward track. Interestingly, in total 41% of the T' within the heat wave region (averaged between 28 and 30 June 2021) formed over the ocean (Supporting Fig. S3), almost exclusively due to poleward moving air parcels (acquiring positive advective T') within ridge R2 (Supporting Anim. 1, Supporting Fig. S3). Between 23 and 26 June the bulk of the air parcels moved onshore, which reduced advective T' of these air parcels due to higher climatological temperatures over land than over ocean during this time of the year (Supporting Anim. 1, between valid times 12 UTC 25 June and 12 UTC 27 June 2021, best visible to the northwest of the heat wave region). The air parcels' descent took place predominantly over land (Supporting Fig. S3), on 27 June and thereafter (Supporting Anim. 1), and was also associated with an average southward movement of the air parcels (i.e., to climatologically warmer regions, Supporting Fig. S4a,b) which further decreased their advective T'. The diabatic T' too built up predominantly over land (Supporting Fig. S3) and mostly within 500 km of the final trajectory location $\boldsymbol{x}(t_X)$ (Extended Data Fig. 2).

Discussion

Neal et al.[1] emphasized the pivotal importance of diabatic heat release in ascending air masses within cyclone C2 for amplifying ridge R2. Our results now further underline the importance of cyclone C2 in the chain of events that ultimately led to the PNW heat wave by showing that T' forming within this ridge ultimately contributed to the PNW heat wave. Moreover, Neal et al.[1] found subsidence and adiabatic warming within the block (i.e., ridge R2) to be of pivotal importance for the extreme PNW near surface temperatures. However, comparing the PNW anomaly composition to the composition of average TX1day anomalies in the PNW region (Extended Data Fig. 9) reveals that the adiabatic T' during the PNW heat wave was not at all unusual for hot extremes in this region, which at first sight appears contradictory to the findings of Neal et al.[1] However, we believe our results are perfectly compatible with theirs, as the subsidence within R2 conceivably happened predominantly in the free troposphere, i.e., above the boundary layer, where it acted to increase the stratification. The near surface air parcels analysed here, on the other hand, did not experience the strong subsidence alluded to by Neal et al. (on average the air parcels experienced a net vertical displacement of only 94 hPa between t_g and t_X). However, it seems plausible that near surface temperature anomalies were nevertheless strongly affected by the free tropospheric subsidence, because it induced a stable stratification above the boundary layer, which suppressed deep convection and ultimately

allowed diabatic T' to accumulate to unprecedented values within the boundary layer (Extended Data Fig. 9).

Finally, it is interesting to compare Lagrangian characteristics of the PNW heat wave to Lagrangian characteristics of the 2003 European and 2010 western Russian heat waves, which were analyzed in detail by Schumacher et al.[3] These authors emphasized the role of upstream sensible (i.e., diabatic) heating of the air for these two heat waves. For the PNW heat wave our results too suggest that remote sources of T' played an important role. However, in the case of the PNW heat wave, T' formed remotely predominantly in the form of advective T'. In contrast to the cases analyzed by Schumacher et al. the large diabatic contributions to the PNW heat wave T' formed rather locally (Extended Data Fig. 2).

References

1. Neal, E., Huang, C. S. Y. & Nakamura, N. The 2021 Pacific Northwest heat wave and associated blocking: meteorology and the role of an upstream cyclone as a diabatic source of wave activity. *Geophys. Res. Lett.* **49**, e2021GL097699 (2022).
2. Rex, D. F. Blocking action in the middle troposphere and its effect upon regional climate. *Tellus* **2**, 196–211 (1950).
3. Schumacher, D. L. *et al.* Amplification of mega-heatwaves through heat torrents fuelled by upwind drought. *Nat. Geosci.* **12**, 712–717 (2019).

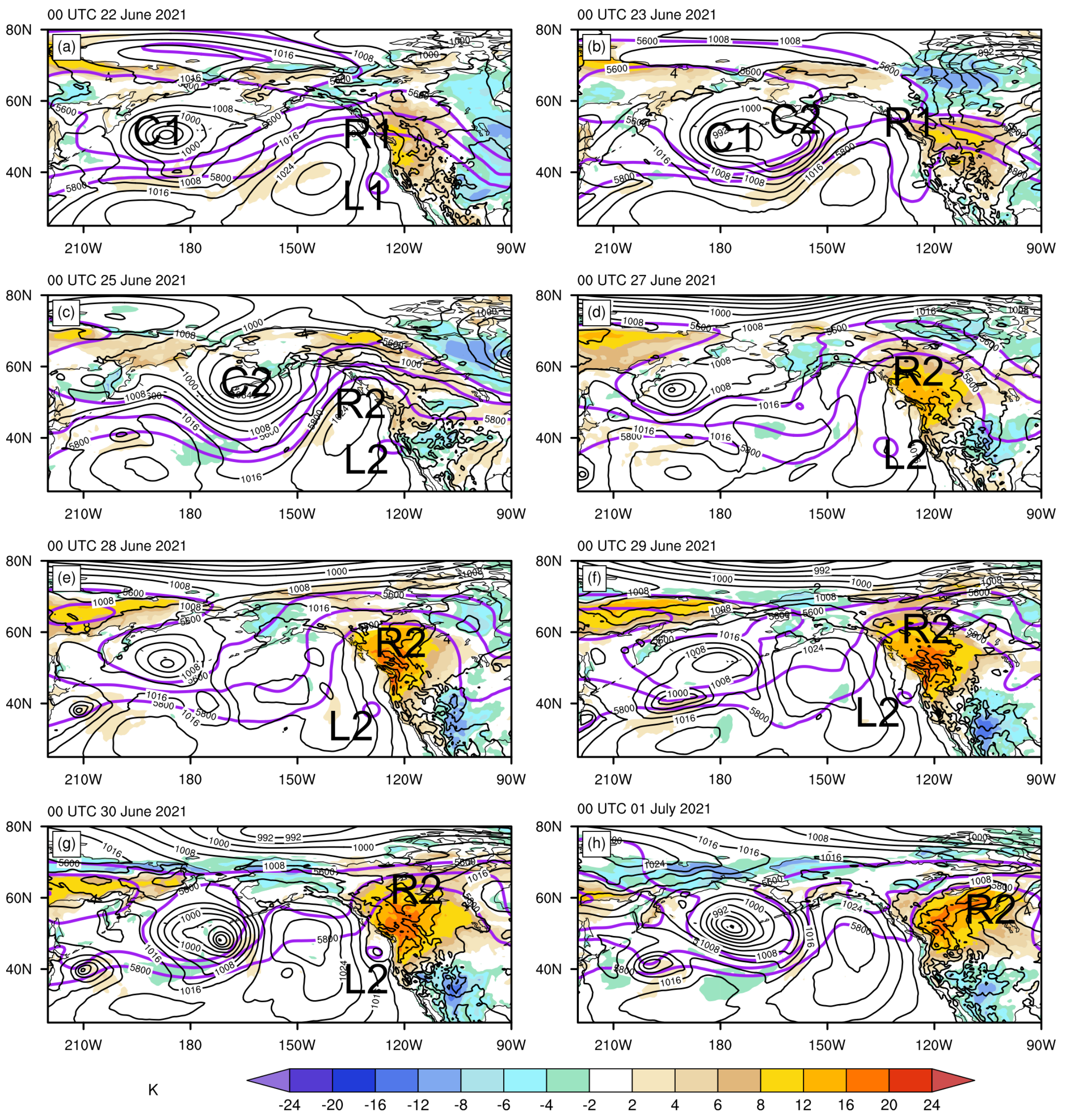

Supporting Figure S1: Synoptic evolution of the PNW heat wave. All panels show T' on the lowest ERA5 model level in shading, sea level pressure (SLP) contours in black and 500 hPa geopotential height contours of 5600, 5700 and 5800 m in purple for the valid time indicated in the top left of each panel. Labels indicate the position of weather systems that are referred to in Supporting Text 1.

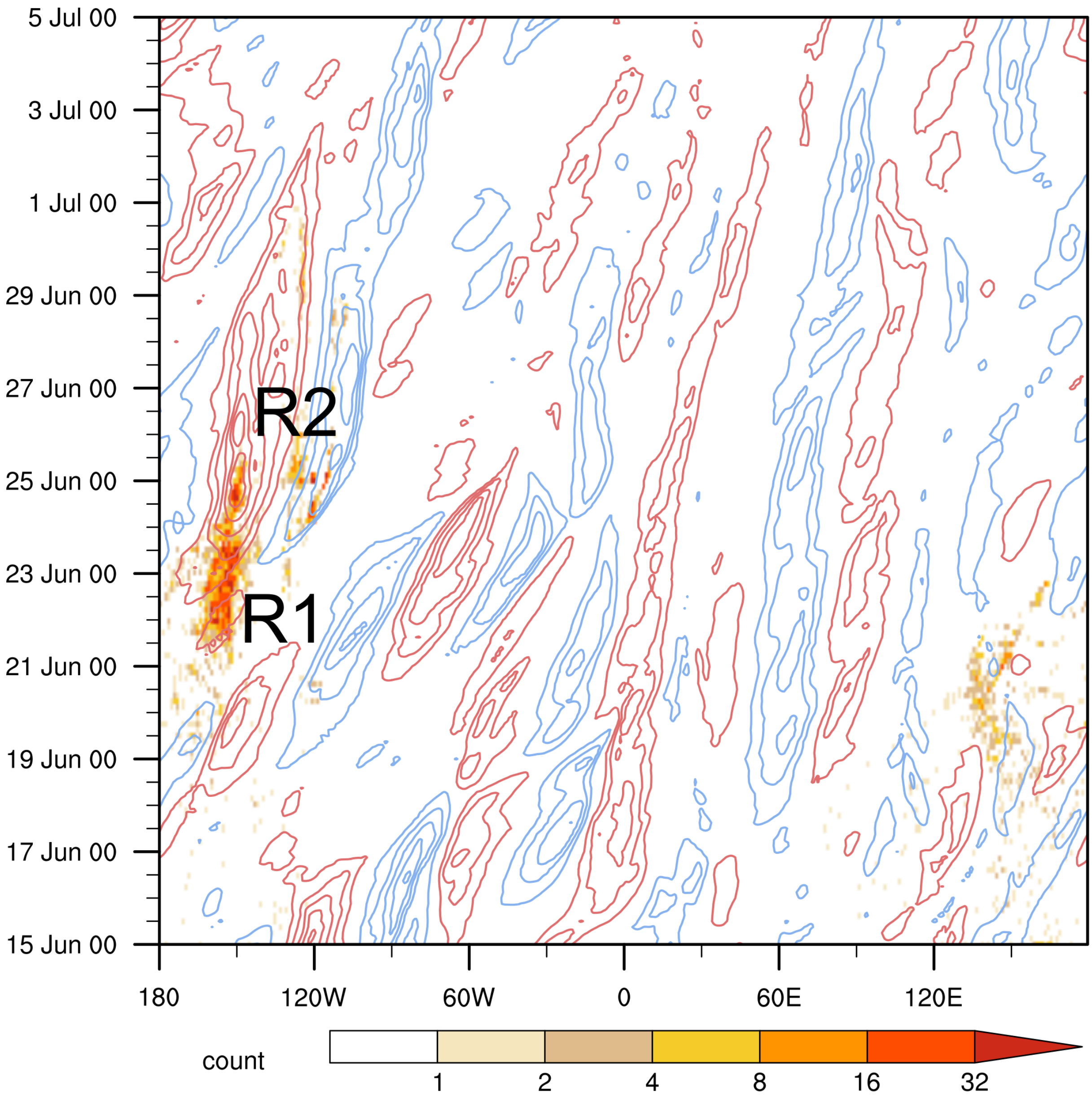

Supporting Figure S2: Longitude-time diagram of genesis locations of anomalies contributing to the PNW heat wave. Red and blue contours show 250 hPa meridional wind, meridionally averaged between 40°N and 60°N of plus and minus 10, 20, 30, 40 and 50 m s^{-1}, respectively. The shading depicts the genesis locations of PNW heat wave temperature anomalies, whereby the genesis locations have been gridded to bins of size 0.5° longitude in the x-direction and 3 hours in the y-direction. Labels identify the ridges R1 and R2 (see Supporting Text 1 for details).

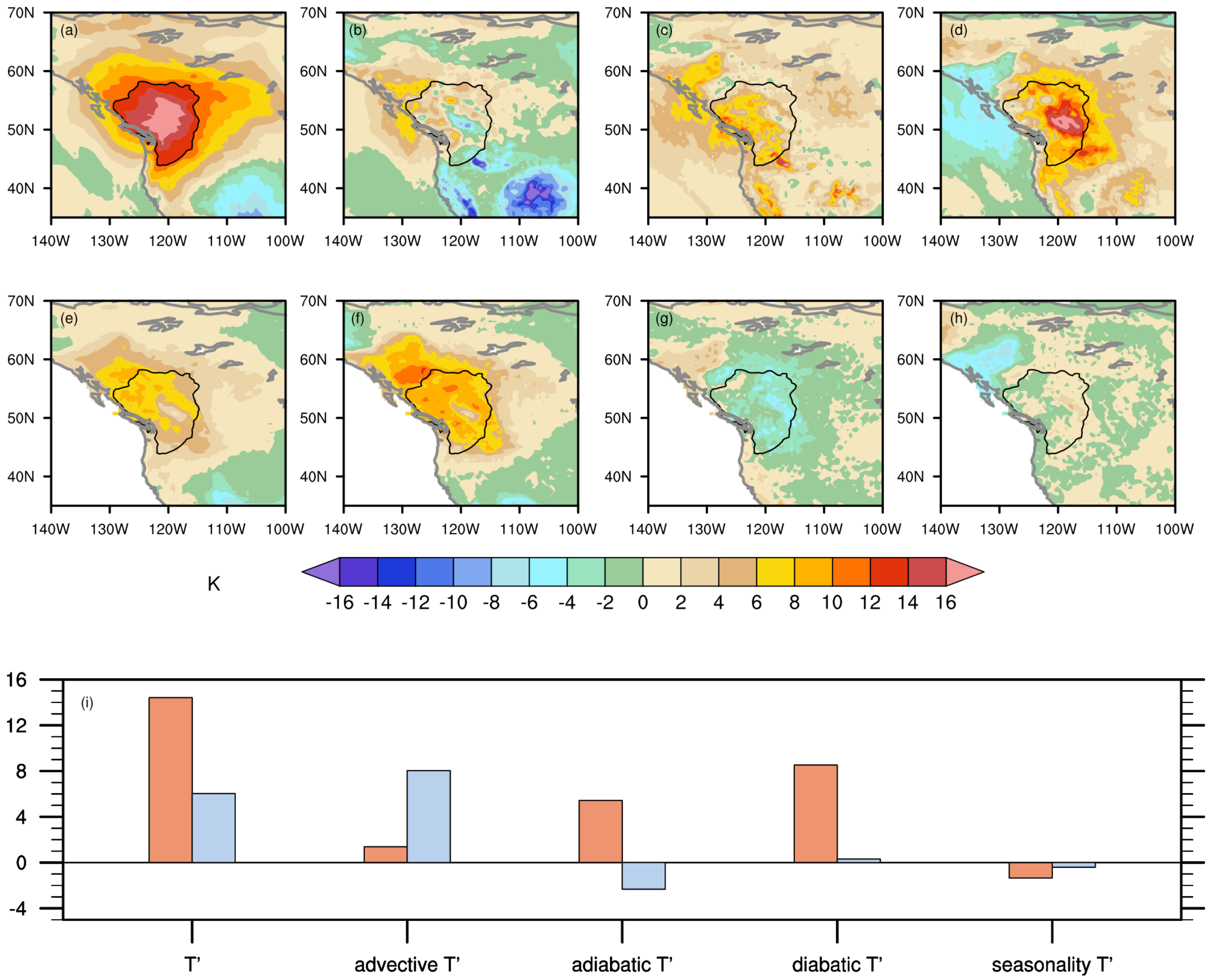

Supporting Figure S3: T' generation over ocean versus over land. (a–d) as in Fig. 1. Panels (e–h) depict for each land grid point the T', advective T', adiabatic T' and diabatic T' generated over the ocean. The quantities have been computed by integrating Eq. (1) along the respective trajectories between t_g and t_o, whereby t_o is the last trajectory time step during which the respective trajectory was located over ocean. For trajectories that were never located over ocean between t_g and t_X a value of zero is assigned for all quantities. Panel (i) shows area weighted averages over the heat wave regions for the total T' in red (i.e., quantities depicted in panels a–d) and for the part of the total T' that was generated over ocean in blue (i.e., quantities depicted in panels e–h).

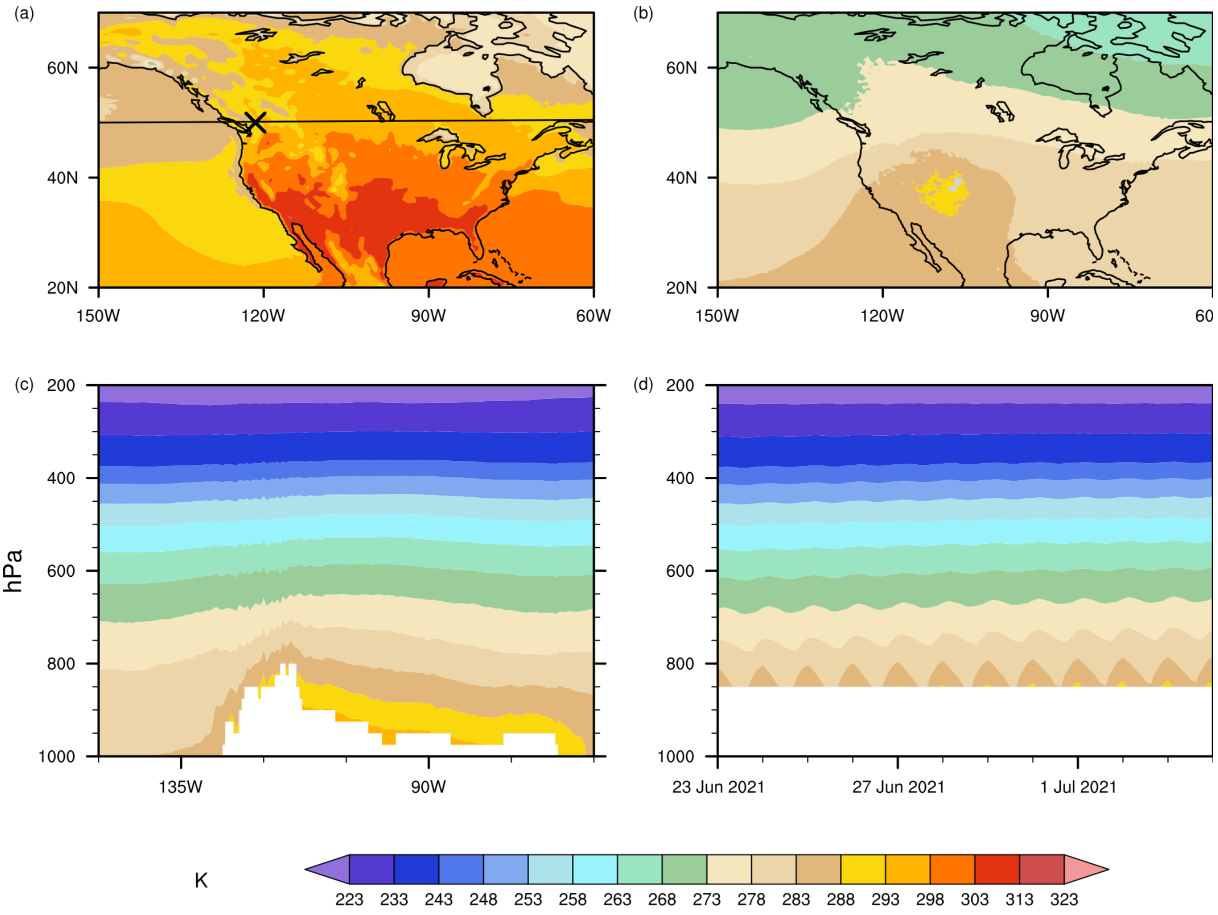

Supporting Figure S4: Illustration of the transient, model-level temperature climatology $\overline{T}$. (a) $\overline{T}$ on the lowest model level for 21 UTC, 29 June 2021, (b) $\overline{T}$ interpolated to 700 hPa for the same time step as in (a), (c) zonal cross-section of $\overline{T}$ at 50°N (black line in (a)), also for 21 UTC, 29 June 2021, and (d) time-height plot of $\overline{T}$ for 121.5°W/50°N (Lytton, Canada, black cross in (a)) for an 12-day period in June/July 2021. Regions where pressure levels intersect the orography are masked out.
