# Supplementary figures and images for: Quantifying the physical processes leading to atmospheric hot extremes at a global scale
Source: Nat Geosci. 2023 Feb 20;16(3):210–6. doi: 10.1038/s41561-023-01126-1 (PMC10005943; doi:10.1038/s41561-023-01126-1)

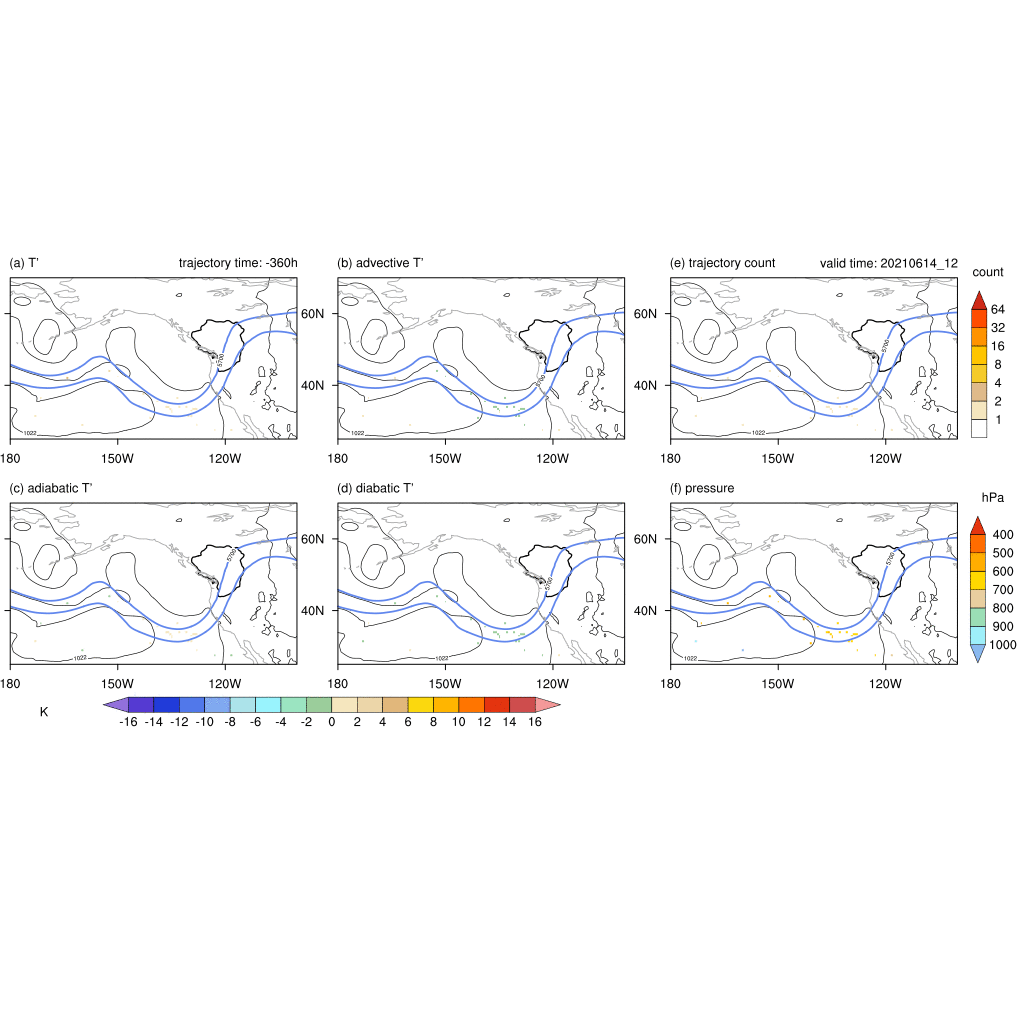

Supplement: Supplementary file 2 — The Lagrangian formation pathway of the June 2021 PNW heat wave. All trajectories contributing to T′ in the heat-wave region (thick black line in all panels) during 28–30 June 2021 have been gridded to a 0.5° latitude by 0.5° longitude grid for all trajectory times after the respective tg. a, T′. b, Advective T′. c, Adiabatic T′. d, diabatic T′. The bottom colour bar is valid for a–d. e, The number of trajectories per 0.5° latitude by 0.5° longitude grid cell. f, The average pressure of all trajectories in the respective grid box. Values in a–d have been averaged across all trajectories per grid box. Sea-level pressure contours (thin black contours) and 500 hPa geopotential height contours of 5,700 m and 5,800 m (blue) are shown at valid times indicated on the top right of e. These valid times correspond to 12:00 utc, 29 June 2021 (the central time step of the 3 d period analysed here) plus the respective trajectory time. [file 41561_2023_1126_MOESM2_ESM.gif]
